# Evaluating Alternatives to Zinc-Bacitracin Antibiotic Growth Promoter in Broilers: Physiological and Meat Quality Responses

**DOI:** 10.3390/ani9121160

**Published:** 2019-12-17

**Authors:** Kwena Thema, Victor Mlambo, Natasha Snyman, Caven Mguvane Mnisi

**Affiliations:** 1 Department of Animal Sciences, Faculty of Natural and Agricultural Science, North-West University, P Bag x2046, Mmabatho 2735, South Africa; themakwena@gmail.com (K.T.); mnisiecm@gmail.com (C.M.M.); 2 Food Security and Safety Niche area, Faculty of Natural and Agricultural Science, North-West University, P Bag x2046, Mmabatho 2735, South Africa; 3 School of Agricultural Sciences, Faculty of Agriculture and Natural Sciences, University of Mpumalanga, P Bag x11283, Mbombela 1200, South Africa; 4 Chemunique (Pty) Ltd., 28 Eagle Lane, Lanseria Business Park, Lanseria 1739, South Africa

**Keywords:** antibiotic growth promoter, blood parameter, broiler, growth performance, meat, non-antibiotic feed additive

## Abstract

**Simple Summary:**

In the Global South, the indiscriminate use of antibiotic growth promoters (AGP) in chicken diets continues to pose a threat to human health. However, since infectious disease burden in these regions is high, withdrawing the use of AGP without alternatives would result in proliferation of infections with serious consequences for food and nutrition security and human health. The effectiveness of different combinations of alternative feed additives (a probiotic, live *Bacillus subtilis*), an organic acid mixture (benzoic and fumaric acids), a protease enzyme, and chelated minerals (Cu, Zn and Mn) was assessed in broiler chickens. When fed on diets containing these alternative feed additives, broilers had similar feed utilization efficiency and growth performance as those fed on an AGP. These findings show that there is potential to replace zinc-bacitracin AGP in broiler diets with feed additives that do not promote antimicrobial resistance and thus deliver safe poultry products.

**Abstract:**

This study evaluated different combinations of a probiotic (*Bacillus licheniformis*), an organic acid mixture (benzoic and fumaric acids), a protease enzyme, and chelated minerals (Cu, Zn, and Mn) as alternatives to zinc-bacitracin antibiotic. Eight hundred Cobb 500 chicks (42.02 ± 2.207 g liveweight) were distributed into 40 pens to which five diets: 1. Commercial broiler diet with no antibiotics (CON); 2. CON + zinc-bacitracin antibiotic (ZnB); 3. CON + chelated minerals + protease enzyme (MinEnz); 4. CON + chelated minerals + protease + organic acids (MinEnzOrg); and 5. CON + chelated minerals + protease + probiotic (MinEnzPro) were allocated. Probiotic, minerals, protease enzyme, and organic acids were included in diets at 0.2 g/kg, 0.3 g/kg, 0.5 g/kg, and 5 g/kg, respectively. Diets promoted a similar feed intake, weight gain, and feed conversion ratio. Birds on MinEnz had the highest basophil content (2.04 × 10^9^/L), while those on ZnB had the highest alanine aminotransferase (8.50 IU/L). Chickens on MinEnz had the heaviest spleens and the largest proventriculi. Meat from CON birds had the highest water holding capacity (22.32%) and cooking losses (27.15%). We concluded that the investigated combinations of feed additives could replace ZnB in broiler diets as they promoted similar growth performance and carcass characteristics.

## 1. Introduction

The profitability of broiler production enterprises is threatened by high feed costs and disease outbreaks. Broilers are susceptible to pathogenic microorganisms such as *Salmonella spp*., *Escherichia coli*, and *Clostridium perfringens* that establish themselves in small intestines, resulting in poor digestion, competition with the host for nutrients [1], high mortality rates, poor meat quality, and food-borne diseases [2]. Consequently, broiler producers often resort to antimicrobial feed additives to prevent diseases and increase yields. Antibiotic growth promoters (AGP) are a prophylaxis against pathogens that result in improved growth performance and production efficiency of broilers [3]. Indeed, zinc-bacitracin (ZnB), a mixture of high molecular weight polypeptides (bacitracin A, B, and C and various minor components) is one such AGP that has growth-promoting effects [1]. However, the continued use of AGPs has received criticism not only because of the risk of endemic bacterial populations developing resistance to the antibiotics, but also because of the presence of antibiotic residues in meat products [4], which pose a threat to human health. These concerns led to the European Union and several other countries to ban the use of AGPs in animal diets [3]. However, in the Global South, the use of AGP is still rife, primarily because the infectious disease burden in these regions is very high while research on potential alternatives to AGP continues to lag behind. Banning AGPs without replacement options will inevitably result in reduced animal performance, poor feed conversion efficiency, and a rise in animal disease incidence. Therefore, it is imperative that the potency of non-antibiotic alternatives, such as chelated trace minerals, organic acids, probiotics, and exogenous feed enzymes, be evaluated. 

These non-antibiotic feed additives are known to negatively affect the growth of pathogenic intestinal bacteria such as *E*. *coli* and *Clostridium perfringens* while improving gut health in chickens [5]. Several studies have been conducted to determine the individual effects of these alternatives on growth indices and their general effect on the microbiota and carcass characteristics of poultry birds [6]. However, when used individually, these additives tend to have weak antimicrobial activities [7]. Chelated trace minerals, organic acids, probiotics, and feed enzymes have the potential to improve bird performance in the absence of antibiotics by increasing resistance to pathogenic bacterial colonization, enhancing mucosal immunity, and aiding nutrient digestion and absorption [5]. Indeed, Pasquet et al. [8] reported that zinc ions (Zn2+) exhibit antimicrobial activity against a number of bacterial and fungal pathogens. On the other hand, probiotics work by producing substances with antimicrobial activity, such as organic acids, and by stimulating immune responses in the host [9]. Combining these individual non-antibiotics is likely to result in the potentiation of antimicrobial activity. Indeed, synergistic antimicrobial activity has previously been demonstrated for organic acids and transition metals such as Mn and Cu [7]. However, there is a paucity of information on the effectiveness of different combinations of chelated minerals, organic acids, enzymes, and probiotic feed additives as alternatives to AGPs in broiler chickens. We hypothesized that combining these feed additives for Cobb 500 broilers would result in physiological and meat quality parameters that are similar to those observed in birds offered the commonly used ZnB antibiotic. Therefore, this study evaluated the effects of combinations of an organic acid mixture (benzoic and fumaric acids), a probiotic (*Bacillus licheniformis*), chelated minerals (copper, zinc, and manganese), and a protease enzyme as alternatives to ZnB on growth performance, blood parameters, and carcass and meat quality traits of Cobb 500 broiler diets. 

## 2. Materials and Methods 

Ethical clearance was obtained from the Animal Research Ethics Committee of the North-West University (Approval number: NWU-00358-17-A9). The study thus conformed to the guidelines on the ethical use of experimental animals.

### 2.1. Study Site and Ingredient Sources

The feeding trial was carried out in spring at Molelwane Research Farm (25.8560° S, 25.6403° E) of the North-West University, South Africa. Temperatures at this time ranged from 7 °C to 28 °C. All feed additives were supplied by Novus International (Centurion, South Africa). The protease enzyme (EC 3.4.21.62) used had a minimum activity of 600,000 U/g. The probiotic consisted of dried *Bacillus licheniformis* PWD-1 (≥4 × 10^9^ CFU/g) mixed with limestone. The minerals (Cu, Zn and Mn) used were chelated with methionine hydroxyl analogue butanoic acid (HMTBa). The organic acid mixture used was a combination of benzoic and fumaric acids blended with HMTBa. All other feed ingredients were purchased from Optifeeds (Mafikeng, South Africa).

### 2.2. Diet Formulation

For the starter, grower, and finisher phases, five isonitrogenous and isocaloric dietary treatments were formulated (Table 1) as follows: 1. A commercial broiler diet without Zn-bacitracin antibiotic growth promoter (CON); 2. a commercial broiler diet with Zn-bacitracin antibiotic growth promoter (ZnB); 3. CON + protease + chelated minerals (MinEnz); 4. CON + protease + chelated minerals + fumaric and benzoic organic acids (MinEnzOrg); and 5. CON + protease + chelated minerals + live *Bacillus* probiotic (MinEnzPro). The inclusion levels of chelated minerals, protease enzyme, organic acids, and probiotic were 0.3 g, 0.5 g, 5 g, and 0.2 g/kg, respectively. The chelated mineral mixture was a composite of 19.3% copper, 36% zinc, and 44.7% manganese by weight.

### 2.3. Chemical Analyses

The formulated diets were analyzed for dry matter, organic matter, crude protein, crude fiber, and crude fat (Table 2) according to the Official Analytical Chemists International methods [10]. Minerals (Ca, Na, Cl) were analyzed according to AgriLasa guidelines [11]. Metabolisable energy and total digestible amino acids were predicted using models from NIRs SpectraStar XL (Unity Scientific, Australia).

### 2.4. Feeding Trial

Eight hundred day-old Cobb 500 chicks were weighed and evenly distributed into 40 pens (1.5 × 1.3 × 2 m each). The pens were the experimental units (20 birds per pen translating into a density of 10 birds per m^2^) and were replicated eight times per dietary treatment. The broiler house was fitted with curtains that were manually rolled up in the morning to allow natural lighting (12 h) and rolled down in the evening. The temperature in the pens ranged from 7 °C to 28 °C, since no artificial heating was provided, except during the starter phase when the temperature was maintained at 32 °C. Humidity ranged from 28–33%, while wind speed ranged from 10–13 km/hour throughout the feeding period. The pens had concrete floors that were covered with wood shavings. At the end of the starter phase on day 13, the birds were fasted for 16 h. On days 14 to 16 only, all birds received a high-protein diet in order to induce stress as described by Kocher et al. [12]. This was designed to induce dysbacteriosis in the gut in order to assess the efficacy of the feed additives. The grower diet was then introduced on day 17 until day 28. Feed and water were offered to the birds *ad libitum* for the entire duration of the experiment (35 days) under natural lighting (13 hours of daylight). Feed intake (FI) and body weight measurements (Explorer EX224, 0.01-g readability (two decimal places) OHAUS Corp, Parsippany, NJ, USA) were taken on a weekly basis and used to calculate feed conversion ratio (FCR). Mortality was recorded on a daily basis and used to calculate percent survival for each replicate pen at the end of the feeding trial. The performance of broilers was also evaluated in terms of the European Broiler Index (EBI) as:

[Survival (%) × Average daily gain (g)]/FCR × 10(1)

### 2.5. Blood Sampling and Analysis

At day 33, two birds from each pen were randomly selected for blood collection by puncturing the brachial vein. Hematological analysis (hematocrit, hemoglobin, erythrocyte, leucocytes, heterophils, lymphocytes, monocytes, eosinophils, and normoblasts) and serum biochemical indices (total protein, albumin, aspartate aminotransferase (AST), alanine transaminase (ALT), sodium, potassium, albumin, urea, calcium, and cholesterol) were analyzed using the IDEXX LaserCyte Hematology Analyzer and an Auto-Analyzer (Hitachi-704, Boehringer Mannheim GmbH, Mannheim, Germany), respectively.

### 2.6. Internal Organs and Carcass Characteristics

At day 35, all birds were starved for 13 h and humanely slaughtered. Weights of internal organs (spleens, empty gizzards, livers without gall bladders, and proventriculi), length of small intestines (measured using a tape measure), and carcass characteristics were determined. Carcasses were immediately weighed to obtain the hot carcass weight (HCW) and thereafter chilled at 4 °C for 24 h and reweighed to obtain the cold carcass weight (CCW). The dressing percentage was determined as the proportion of HCW to slaughter weight. All weights were taken using a weighing scale (Explorer EX224, 0.01-g readability (two decimal places), supplied by OHAUS Corp (Parsippany, NJ, USA).

### 2.7. Meat Quality Analysis

Meat pH was measured immediately after slaughter using a Corning Model 4 pH-temperature meter (Corning Glass Works, Medfield, MA, USA) equipped with an Ingold spear-type electrode (Ingold Messtechnik AG, Udorf, Switzerland). The pH meter was calibrated using pH 10.0, 7.0 and 4.0 buffers at 25 °C. Meat colour (L* = lightness, a* = redness, b* = yellowish) was determined 24 h post-mortem using a Minolta colour-guide (Spectrophotometer CM 2500c, Konika Minolta, Osaka, Japan). The water holding capacity (WHC) was determined as the amount of water expressed from fresh breast meat sample (8–16 g) held under pressure (60-kg pressure) using the filter-paper press method. The water from the sample was absorbed by a pre-weighed filter paper and calculated as a proportion of the initial weight. Drip loss (%) was determined as the loss in weight of a meat sample suspended in bottle and stored in a cold room at 4 °C for 72 h. For cooking losses, pre-weighed breast samples were placed in an oven set at 130 °C for 20 min. The losses were calculated as the difference between the final (cooked) and initial weights of the breasts and expressed as a proportion of initial weight. After cooking, cylindrical samples (12.5-mm core diameter) of breast muscle were sheared perpendicular to the fibre direction using a Warner-Bratzler shear device mounted on a Universal Instron apparatus (cross head speed = 200 mm/min, one shear in the center of each core). The reported value represented the average shear force measurements in Newtons.

### 2.8. Statistical Analysis

Data for each measured parameter were tested for normality using the ‘Normal’ option in the Proc Univariate statement. Weekly feed intake, weight gain, and FCR data were analyzed using the repeated measures analysis [13]. The following statistical linear model was employed:

(2) $$Y_{ijk}=\mu +T_{i}+W_{j}+{\left(T\times W\right)}_{ij}+E_{ijk}$$

where *Y_ijk_* = dependent variable, *µ* = population mean, *T_i_* = effect of dietary treatments, *W_j_* = effect of week, (*T × W*)*_ij_* = effect of interaction between dietary treatments and week, and *E_ijk_* = random error associated with observation *ijk*, assumed to be normally and independently distributed. 

Overall FI, body weight gain (BWG), FCR, blood parameters, size of internal organs, and carcass and meat quality data were analyzed using the general linear models (GLM) procedure of SAS version 9.4 [13] according to the following linear statistical model:

(3) $$Y_{ij}=\mu +T_{i}+E_{ij}$$

where *Y_ij_* = dependent variable, *µ* = population mean constant common to all observations, *T_i_* = effect of diet, and *E_ij_* = random error associated with observation *ij*, assumed to be normally and independently distributed. For all tests, the level of significance was set at *p* ≤ 0.05.

## 3. Results

### 3.1. Feed Intake, Growth Performance, and Blood Parameters

Repeated measures analysis revealed no significant week × diet interaction effect on feed intake, weight gain, and FCR. Dietary treatments had no effect (*p* > 0.05) on overall feed intake, overall weight gain, overall FCR, survival, and European Broiler Index, as shown in Table 3. 

Table 4 shows that for hematological parameters, diets influenced (*p* < 0.05) basophils only. Whereas for serum biochemical indices, diets affected ALT levels only. Birds on MinEnz had the highest basophil content (2.04 × 10^9^/L) compared to all the other treatments. Diet ZnB promoted higher ALT (8.50 IU/L) than diet MinEnz (2.25 IU/L). Birds on diet ZnB had statistically similar ALT levels to those in CON, MinEnzOrg, and MinEnzPro. 

### 3.2. Internal Organs, Carcass Characteristics, and Bone Measurements

Table 5 shows that diets had no effect (*p* > 0.05) on the weights of the empty gizzard, liver, and small intestines, but influenced spleen and proventriculus weights. Chickens on MinEnz had the heavier spleens (3.81 g) compared to those on diets CON and MinEnzPro. There were no differences among broilers on ZnB, MinEnz and MinEnzOrg in terms of spleen weights. Birds on MinEnz had the heavier proventriculi (12.63 g) compared to those on diets ZnB and MinEnzOrg. There were no differences between birds on CON, MinEnz, and MinEnzPro diets in terms of proventriculi weights. No dietary influences (*p* > 0.05) were observed on slaughter weights, HCW, CCW, breasts, and dressing out percentage of broiler chickens (Table 5). 

### 3.3. Meat Quality Traits 

No dietary effects (*p* > 0.05) were observed on meat pH, lightness, redness, yellowness, drip loss, and shear force (Table 6). However, there were dietary effects (*p* < 0.05) on WHC and cooking loss. Meat from birds on CON had higher (*p* < 0.05) WHC (22.32%) compared to meat from the other diets. Meat from birds on CON had higher cooking losses (27.15%) than meat from birds on diets MinEnzOrg and MinEnzPro. 

## 4. Discussion

### 4.1. Growth Performance and Blood Parameters

Chelated minerals, protease enzyme, organic acids, and probiotics have been shown in some studies to improve the physiological responses and meat quality parameters of poultry birds [2] when used separately. Examples include Mnisi and Mlambo [14], who reported that the inclusion of protease enzyme did not improve the performance of Japanese quails, while Atela et al. [15] concluded that the inclusion of a multi-strain probiotic through drinking water enhances feed utilization and meat quality traits of indigenous chickens. However, despite the potential for synergistic effects, few studies have evaluated these additives in combination. In this study, combinations of these alternative additives did not alter feed intake, survival, growth performance and EBI of the birds. The high EBI values across all the diets indicate that body weight gain of broilers was uniform and the flock was in good health. Vale et al. [16] also reported that dietary inclusion of antibiotics, probiotics and organic acids had no effect on weight gain and FCR. Generally, most reports on the effects of feed additives on broiler performance are inconsistent, which can be attributed to a number of factors, such as the environment, management, nutrition, additive types, dosage, and bird characteristics (age, species, stage of production) that ultimately affect responses to feed additives [17]. For example, Angel et al. [18] reported that broilers grown under favorable conditions, in the absence of stress and diseases, do not benefit from the inclusion of probiotics. Similarly, Anderson et al. [19] further stated that birds that are well-nourished and reared under optimal conditions (clean environment and at moderate stocking density) do not respond positively to growth promoters such as organic acids, probiotics, or antibiotics. In an attempt to address this, broilers in this study were deliberately reared under moderately stressful conditions through short-term dietary manipulation and long-term higher-than-normal stocking density. Under these stressful rearing conditions, results showed that the ZnB antibiotic had similar effects on broiler performance as the alternative feed additives.

Nutritional factors are known to affect intermediary metabolism, resulting in changes in plasma metabolite levels in poultry [20,21]. Thus, it is imperative to monitor the pathophysiological status of the birds in studies that may alter nutritional status as well as immune responses. Of all the measured hematological parameters, only basophil levels were altered by dietary inclusion of feed additives, with birds receiving the diet containing protease and chelated minerals (MinEnz) exhibiting basophilia. It is not clear why birds on this diet had the highest basophils level, considering the fact that the other diets had similar basophil levels as the control diet. It has been suggested that avian basophils are associated with stress responses in birds [22], with a significant increase in basophils being observed in stressed birds. Feed restriction is one of the stressors that has also been shown to produce significant basophilia in broilers. However, throughout the feeding trial and at the time of blood sampling, all birds were handled in the same way, thus ruling out stress as a cause for observed basophilia. A study conducted by Cetin et al. [23] found that addition of a probiotic (live *Bacillus* spp.) to the diet caused an increase in both hemoglobin concentration and hematocrits. However, in this study, no such changes were observed, in line with the findings of Baidya et al. [24], who reported that inclusion of probiotics and antibiotics in broiler diets did not affect hemoglobin concentration. The lack of differences in the other blood indices suggests that the alternative feed additives have the potential to replace zinc bacitracin antibiotics, even under the stressful rearing conditions of the current study. In addition, the total protein and cholesterol levels from this study confirm the findings from Hernández et al. [25] that organic acid and probiotic inclusion do not alter these parameters in broiler chickens.

### 4.2. Internal Organs, Carcass Traits, and Bone Measurements

The lack of dietary influence on the size of internal organs, with the exception of the spleen, has also been reported in other studies [26,27]. In this study, the weight of the spleen was higher in broilers fed the protease enzyme and chelated minerals, a result also reported by Award et al. [28]. It is not clear why birds on MinEnz had heavier spleen weights than those on negative control, even though the spleen is the main secondary lymphoid organ in a chicken and responsible for regulating immune responses [29]. Diets had no effect on hot carcass weight, cold carcass weight, and dressing percentage of the broilers. Similar findings were reported on carcass yields of birds fed dietary organic acids [30], a probiotic [16], and a protease enzyme [15]. In contrast, Pelicano et al. [31] reported lower carcass weights in birds given probiotics (live *Bacillus*)-containing diets. 

### 4.3. Meat Quality Measurements

Meat pH affects meat quality parameters such as flavor, tenderness, shelf-life, and color. The meat pH values in the current study ranged from 7.01 to 7.17 and there were no dietary differences observed. The recorded meat pH values are higher than previously reported and we were unable to explain this variation, which could be an error in measurement. Organic acids exert antimicrobial effects on the feed before consumption and upon ingestion in the crop, gizzard and intestine [32], but do not seem to affect meat pH. Diets had no effect on meat quality, which is in line with the findings of Li et al. [33]. Inconsistent meat quality results have been reported when probiotics have been used in chicken diets. Some scholars reported positive probiotic effects [34], while others recorded no beneficial effects [35]. In this study, diets had an effect on cooking loss and WHC. Drip loss and WHC are some of the most important indicators of meat quality because some nutrients may be lost in the exudate, which may have a negative effect on the tenderness, flavor, and juiciness of the meat [36]. The WHC of chicken meat products is also related to final carcass yield that affect profitability [37]. Water loss reduces the overall weight of the product, which contributes to financial loss through reduction in the weight of the salable product. Chelated mineral supplementation did not show any effects on meat quality, which was in agreement with findings by Lu et al. [38], who reported that supplementing Mn does not affect meat traits. 

## 5. Conclusions

We concluded that the inclusion of a combination of alternative feed additives in broiler diets resulted in similar feed utilization efficiency, survival, growth performance, and carcass characteristics as the diet containing the ZnB antibiotic growth promoter. Blood indices were observed to be within the range for healthy broilers for all the dietary treatments.

## Figures and Tables

**Table 1:** Ingredient composition (%) of the dietary treatments at the starter, grower, and finisher phases.

Ingredients	Dietary Treatments ^1^														
	Starter (1–13 Days)	Grower (14–28 Days)					Finisher (29–35 Days)								
	CON	ZnB	MinEnz	MinEnzOrg	MinEnzPro	CON	ZnB	MinEnz	MinEnzOrg	MinEnzPro	CON	ZnB	MinEnz	MinEnzOrg	MinEnzPro
Maize	42.71	42.71	42.71	42.71	42.71	46.37	46.37	46.37	46.37	46.37	51.51	51.51	51.51	51.51	51.51
Soy oilcake	16.0	16.0	16.0	16.0	16.0	16.0	16.0	16.0	16.0	16.0	13.0	13.0	13.0	13.0	13.0
Sunflower oilcake	5.0	5.0	5.0	5.0	5.0	0	0	0	0	0	0	0	0	0	0
Wheat bran	32.5	32.5	32.5	32.5	32.5	34.5	34.5	34.5	34.5	34.5	32.5	32.5	32.5	32.5	32.5
Limestone	1.1	1.1	1.1	1.1	1.1	1.0	1.0	1.0	1.0	1.0	0.95	0.95	0.95	0.95	0.95
Mono-calcium phosphate	1.1	1.1	1.1	1.1	1.1	0.95	0.95	0.95	0.95	0.95	0.8	0.8	0.8	0.8	0.8
Fine-Salt	0.15	0.15	0.15	0.15	0.15	0.2	0.2	0.2	0.2	0.2	0.2	0.2	0.2	0.2	0.2
Lysine	0.39	0.39	0.39	0.39	0.39	0.28	0.28	0.28	0.28	0.28	0.26	0.26	0.26	0.26	0.26
Methionine	0.29	0.29	0.29	0.29	0.29	0.24	0.24	0.24	0.24	0.24	0.22	0.22	0.22	0.22	0.22
Threonine	0.15	0.15	0.15	0.15	0.15	0.1	0.15	0.1	0.1	0.1	0.1	0.1	0.1	0.1	0.1
Axtra^®^ PHY (Phytase)	0.01	0.01	0.01	0.01	0.01	0.01	0.01	0.01	0.01	0.01	0.01	0.01	0.01	0.01	0.01
Vitamin premix	0.25	0.25	0.25	0.25	0.25	0.1	0.1	0.1	0.1	0.1	0.2	0.2	0.2	0.2	0.2
Zinc-Bacitracin 15%	0	0.05	0	0	0	0	0.05	0	0	0	0	0.05	0	0	0
Monensin 20%	0.05	0.05	0.05	0.05	0.05	0.05	0.05	0.05	0.05	0.05	0.05	0.05	0.05	0.05	0.05
Sodium bicarbonate	0.3	0.3	0.3	0.3	0.3	0.2	0.2	0.2	0.2	0.2	0.2	0.2	0.2	0.2	0.2
Organic acids	0	0	0	0.50	0	0	0	0	0.50	0	0	0	0	0.5	0
Probiotic	0	0	0	0	0.02	0	0	0	0	0.02	0	0	0	0	0.02
Chelated minerals	0	0	0.03	0.03	0.03	0	0	0.03	0.03	0.03	0	0	0.03	0.03	0.03
Protease	0	0	0.05	0.05	0.05	0	0	0.05	0.05	0.05	0	0	0.05	0.05	0.05

^1^ Dietary treatments: CON = a commercial broiler diet without Zn-bacitracin antibiotic growth promoter; ZnB = a commercial broiler diet with Zn-bacitracin antibiotic growth promoter; MinEnz = CON + protease + chelated minerals; MinEnzOrg = CON + protease + chelated minerals + fumaric and benzoic organic acids; MinEnzPro = CON + protease + chelated minerals + live *Bacillus* probiotic.

**Table 2:** Chemical composition (%, unless stated otherwise) of starter, grower, and finisher diets for broilers.

Ingredients	Starter (1–13 days)	Grower (14–28 days)	Finisher (29–35 days)
Metabolisable energy (MJ/Kg)	10.1	9.8	9.9
Dry matter	87.7	87.6	87.6
Organic matter	82.0	82.2	82.6
Crude protein	18.2	16.8	15.4
Crude fat	3.26	3.36	3.42
Crude fibre	6.11	5.36	5.14
Calcium	0.81	0.73	0.66
Sodium	0.14	0.14	0.14
Chlorine	0.25	0.25	0.25
Digestible lysine	0.76	0.85	0.98
Digestible methionine	0.41	0.44	0.51
Digestible threonine	0.54	0.58	0.68
Digestible tryptophan	0.12	0.14	0.15
Digestible arginine	0.75	0.83	0.91
Digestible isoleucine	0.50	0.55	0.60

**Table 3:** The effects of dietary treatments on feed intake, weight gain, feed conversion ratio, survival and European broiler index of chickens.

Parameters	Dietary Treatments ^1^					
	CON	ZnB	MinEnz	MinEnzOrg	MinEnzPro	SEM
Weight gain (g)	2682	2823	2767	2807	2807	83.0
Feed intake (g)	3299	3165	3275	3310	3313	71.4
Feed conversion ratio	1.23	1.14	1.18	1.17	1.18	0.03
Survival (%)	89.4	89.4	91.3	91.9	87.5	1.51
European Broiler Index ^2^	552.4	632.5	611.7	629.9	594.7	42.45

^1^ Dietary treatments: CON = a commercial broiler diet without Zn-bacitracin antibiotic growth promoter; ZnB = a commercial broiler diet with Zn-bacitracin antibiotic growth promoter; MinEnz = CON + protease + chelated minerals; MinEnzOrg = CON + protease + chelated minerals + fumaric and benzoic organic acids; MinEnzPro = CON + protease + chelated minerals + live *Bacillus* probiotic; ^2^ European Broiler Index = [Survival (%) × Average Daily Gain (g/bird/day)]/Feed Conversion Ratio × 10.

**Table 4:** The effect of dietary treatments on blood parameters of broiler chickens.

Parameters	Dietary Treatments ^1^					SEM
	CON	ZnB	MinEnz	MinEnzOrg	MinEnzPro	
*Hematological parameters*						
Erythrocytes (×10^12^/L)	2.68	2.39	2.59	2.63	2.68	0.1
Hemoglobin (g/dL)	9.52	8.33	9.37	9.37	9.57	0.32
Hematocrits (g/dL)	0.37	0.31	0.34	0.34	0.36	0.01
Leucocytes (×10^9^/L)	26.5	22.7	27.3	22.1	23.3	4.06
Heterophils (×10^9^/L)	1.18	0.89	0.62	0.91	0.52	0.38
Lymphocytes (×10^9^/L)	15.03	16.82	22.5	18.53	19.49	2.85
Monocytes (×10^9^/L)	0.66	1.27	0.73	0.95	0.79	0.38
Eosinophils (×10^9^/L)	1.85	1.31	1.36	1.34	1.57	0.6
Basophils (×10^9^/L)	0.85^a^	0.51^a^	2.04^b^	0.12^a^	0.95^a^	0.36
*Serum biochemical parameters*						
ALT ^2^ (IU/L)	5.75 ^a,b^	8.5 ^b^	2.25 ^a^	3.37 ^a,b^	2.87 ^a,b^	1.58
AST ^3^ (IU/L)	391.3	336.9	380.6	358.5	265.8	36.26
Total protein (g/L)	33.37	29.75	29.5	31.75	31	1.14
Sodium (mmol/L)	147.3	144.5	144	144.3	144.3	1.25
Albumin (g/L)	13.75	12.80	12.62	13.75	13.87	0.44
Urea (mmol/L)	0.39	0.33	0.2	0.37	0.31	0.05
Calcium total (mmol/L)	2.66	2.57	2.71	2.81	2.71	0.12
Cholesterol (mmol/L)	3.73	3.61	3.46	3.72	3.72	0.14

^a,b^ In a row, means with common superscripts do not differ (*p* > 0.05); ^1^ Dietary treatments: CON = a commercial broiler diet without Zn-bacitracin antibiotic growth promoter; ZnB = a commercial broiler diet with Zn-bacitracin antibiotic growth promoter; MinEnz = CON + protease + chelated minerals; MinEnzOrg = CON + protease + chelated minerals + fumaric and benzoic organic acids; MinEnzPro = CON + protease + chelated minerals + live *Bacillus* probiotic; ^2^ ALT = alanine transaminase; ^3^ AST = aspartate aminotransferase.

**Table 5:** The effects of dietary treatments on weights of internal organs, length of small intestines, and carcass traits (g, unless stated otherwise) of broilers.

Parameters	Dietary Treatments ^1^					
	CON	ZnB	MinEnz	MinEnzOrg	MinEnzPro	SEM
Spleen	2.12 ^a^	2.75 ^a,b^	3.81 ^b^	2.67 ^a,b^	2.32 ^a^	0.32
Empty gizzard	32.88	34.12	35.25	35.13	33.86	2.18
Liver	42.63	40.75	43.5	45.25	42.14	2.17
Proventriculus	11.63 ^a,b^	9.63 ^a^	12.63 ^b^	10.13 ^a^	10.43 ^a,b^	0.61
Small intestine (cm)	155.75	147.44	145.81	155.57	147.64	4.43
Final body weight	2726.2	2804.2	2773.1	2850.7	2804.3	84.6
Hot carcass weight	1716.5	1570.5	1590.2	1673.1	1619.6	55.13
Cold carcass weight	1684.5	1548.5	1560.6	1620.6	1631.3	53.13
Breast	371.75	365.62	342.75	378	417	26.82
Dressing (%)	63.95	57.22	56.5	61.67	62.34	3.05

^a,b^ In a row, means with common superscripts do not differ (*p* > 0.05); ^1^ Dietary treatments: CON = a commercial broiler diet without Zn-bacitracin antibiotic growth promoter; ZnB = a commercial broiler diet with Zn-bacitracin antibiotic growth promoter; MinEnz = CON + protease + chelated minerals; MinEnzOrg = CON + protease + chelated minerals + fumaric and benzoic organic acids; MinEnzPro = CON + protease + chelated minerals + live *Bacillus* probiotic.

**Table 6:** The effects of dietary treatments on breast meat quality traits of broiler chickens.

Parameters	Dietary Treatments ^1^					
	CON	ZnB	MinEnz	MinEnzOrg	MinEnzPro	SEM
pH	7.01	7.16	7.11	7.1	7.11	0.05
Lightness (L*)	69.83	67.24	60.04	68.27	66.95	3.16
Redness (a*)	2.17	3.17	1.35	2.14	2.22	0.67
Yellowness (b*)	14.04	13.79	11.69	13.67	14.28	0.89
WHC^2 2^ (%)	22.32 ^b^	15.92 ^a^	15.74 ^a^	12.53 ^a^	12.21 ^a^	1.34
Drip loss (%)	12.75	14.29	13.61	11.88	19.31	3.54
Cooking loss (%)	27.15 ^b^	26.03 ^a,b^	23.79 ^a,b^	21.45 ^a^	21.65 ^a^	1.35
Shear force (N)	10.39	8.64	9.16	9.23	9.29	1.51

^a,b^ In a row, means with common superscripts do not differ (*p* > 0.05); ^1^ Dietary treatments: CON = a commercial broiler diet without Zn-bacitracin antibiotic growth promoter; ZnB = a commercial broiler diet with Zn-bacitracin antibiotic growth promoter; MinEnz = CON + protease + chelated minerals; MinEnzOrg = CON + protease + chelated minerals + fumaric and benzoic organic acids; MinEnzPro = CON + protease + chelated minerals + live *Bacillus* probiotic; ^2^ WHC = Water holding capacity.
